# USING DATA TO DRIVE POLICY: AN EXAMPLE OF A STATE–UNIVERSITY PARTNERSHIP TO ADVANCE AGE-FRIENDLY COMMUNITIES

**DOI:** 10.1093/geroni/igae098.1027

**Published:** 2024-12-31

**Authors:** Howard Degenholtz, Steven Albert, Keri Kastner, Heather Mentch, Kevin Hancock, Gabrielle Szymanski, Nathan Lampenfeld

**Affiliations:** University of Pittsburgh, Pittsburgh, Pennsylvania, United States; University of Pittsburgh, Pittsburgh, Pennsylvania, United States; University of Pittsburgh, Pittsburgh, Pennsylvania, United States; University of Pittsburgh, Pittsburgh, Pennsylvania, United States; Pennsylvania Department on Aging, Harrisburg, Pennsylvania, United States; Pennsylvania Department on Aging, Harrisburg, Pennsylvania, United States; Pennsylvania Department on Aging, Harrisburg, Pennsylvania, United States

## Abstract

The Pennsylvania Department on Aging (PDA) is developing a “Master Plan for Older Adults” called “Aging Our Way.” The plan will help guide policies and programs for the next 10 years. Agency leadership turned to the University of Pittsburgh to conduct a statewide needs assessment survey as part of a broad stakeholder engagement process that involved community meetings in all 67 counties. The University conducted a statewide telephone survey drawing on the Age-Friendly Community model. A sample of adults age 60 and older was stratified into eight regions to assure that the entire state was represented. To reach non-English speaking adults and disabled adults who might not respond by telephone, a web-based version of the survey was developed and translated into Chinese, Spanish and Russian and distributed via email to stakeholder groups. Paper versions in were mailed to community-based organizations to reach individuals who were not able to access the internet. The telephone survey results (n=900) were weighted to match the 2021 American Community Survey. The web-based survey results (n=6950) are reported separately. Findings were presented to the Long-Term Care Council, a stakeholder group that is convened by the PDA to advise on policy. The Council was formed into several subcommittees that addressed different policy topics (e.g., housing, transportation). The draft report developed by the Council was shared with the University team to develop objectives that could be measured by repeating the survey. The overall collaboration between the University and the PDA led to a strong, empirically based report.

